# Enhancement of anti-DIII antibodies by the C3d derivative P28 results in lower viral titers and augments protection in mice

**DOI:** 10.1186/1743-422X-7-95

**Published:** 2010-05-12

**Authors:** Matthew D Dunn, Shannan L Rossi, Donald M Carter, Matthew R Vogt, Erin Mehlhop, Michael S Diamond, Ted M Ross

**Affiliations:** 1 Center for Vaccine Research, University of Pittsburgh, 9047 Biomedical Science Tower 3, 3501 Fifth Avenue, Pittsburgh, PA 15261, USA; 2 Department of Microbiology and Molecular Genetics, University of Pittsburgh, 9047 Biomedical Science Tower 3, 3501 Fifth Avenue, Pittsburgh, PA 15261, USA; 3 Departments of Medicine, Molecular Microbiology, Pathology & Immunology, Washington University School of Medicine, 660 South Euclid Ave., Box 8051, St. Louis, MO 63110, USA

## Abstract

Antibodies generated against West Nile virus (WNV) during infection are essential for controlling dissemination. Recent studies have demonstrated that epitopes in all three domains of the flavivirus envelope protein (E) are targets for neutralizing antibodies, with determinants in domain III (DIII) eliciting antibodies with strong inhibitory properties. In order to increase the magnitude and quality of the antibody response against the WNV E protein, DNA vaccines with derivatives of the WNV E gene (full length E, truncated E, or DIII region, some in the context of the pre-membrane [prM] gene) were conjugated to the molecular adjuvant P28. The P28 region of the complement protein C3d is the minimum CR2-binding domain necessary for the adjuvant activity of C3d. Delivery of DNA-based vaccines by gene gun and intramuscular routes stimulated production of IgG antibodies against the WNV DIII region of the E protein. With the exception of the vaccine expressing prM/E given intramuscularly, only mice that received DNA vaccines by gene gun produced protective neutralizing antibody titers (FRNT_80 _titer >1/40). Correspondingly, mice vaccinated by the gene gun route were protected to a greater level from lethal WNV challenge. In general, mice vaccinated with P28-adjuvated vaccines produced higher IgG titers than mice vaccinated with non-adjuvanted vaccines.

## Introduction

West Nile virus (WNV) is a single-stranded positive polarity enveloped RNA virus and member of the Flavivirus genus of the *Flaviviridae *family. The genome (11 kb) encodes for three structural proteins (Capsid [C] [1], pre-membrane [prM] that is cleaved to form a mature membrane [M] [2] and Envelope [E] [1]) and seven nonstructural gene products (NS1, 2A, 2B, 3, 4A, 4B and 5). WNV is transmitted by mosquitoes and causes morbidity and mortality in birds, horses, and humans. Since 1999, there have been over 29,000 cases that reached clinical attention and resulted in greater than a thousand deaths http://www.cdc.gov/ncidod/dvbid/westnile/surv&control.htm within the United States as reported to the Centers for Disease Control and Prevention. As the geographic distribution of this virus continues to expand, naïve human populations are put at greater risk, making the need for a licensed vaccine and/or antiviral treatment pressing [3].

The host immune response is critical for limiting virus spread and disease. Results from genetically engineered mice indicate that both the innate (*e.g*., interferon) and the adaptive (B and T cells) immune responses control WNV infection [4]. The production of antibodies is essential to protection against WNV infection [5], and passive antibody transfer of anti-WNV neutralizing antibodies can prevent or treat lethal infection [6]. The primary target of the neutralizing antibody response is the E protein, which is the most accessible structural glycoprotein on the surface of the virion [7]. Structural analysis of the soluble ectodomain of flavivirus E proteins reveals three domains [8,9]. Domain I is an 8-stranded β-barrel that participates in the conformational changes associated with the acidification of the endosome. Domain II, which contains 12 β-strands, has important roles in dimerization, trimerization, and virus-mediated fusion [10-12]. Domain III adopts an immunoglobulin-like fold that contains the most distal projecting loops on the mature virion [13,14], and has been hypothesized to contain a binding site for cell attachment [15]. Even though neutralizing antibodies are generated against epitopes in all three domains, many highly neutralizing antibodies cluster to epitopes in DIII [16].

Our laboratory and others have demonstrated that the fusion of C3d to an antigen results in enhanced immunogenicity of the fused antigen [5,16,17,19,21,24,31,32,35,36]. C3d is the final degradation product of the third component of complement (C3). The most commonly proposed mechanisms for C3d adjuvanticity involves C3d binding to the complement receptor 2 (CR2) that is located on the surface of follicular dendritic cells (FDC), B cells, and T cells in many species (for review, see [17]). C3d stimulates antigen presentation by FDCs and helps to maintain immunological B cell memory. On B cells, C3d interacts with CR2, CD19 and CD81 surface molecules. CD19 has a long intracellular tail that triggers a signaling cascade that results in cell activation and proliferation. Simultaneous ligation of CR2 by C3d and surface immunoglobulin by antigen activates two signaling pathways that synergize to activate B cells, thereby leading to enhanced antibody secretion against the fused antigen. Multimers of a 28 amino acid peptide of C3d (P28), which contains the predicted minimum CR2 binding domain, have been demonstrated to have similar adjuvant properties as the entire C3d molecule [18]. The P28 molecule is ~9% the size of the entire C3d molecule and therefore, is an attractive adjuvant to elicit enhanced B cell responses to a vaccine antigen.

Currently, there are no effective anti-WNV treatments and there are no Food and Drug Administration (FDA)-licensed vaccines for humans. The FDA has approved a WNV vaccine for horses and other exotic animals, based upon a formalin-inactivated killed virus (WNV Innovator™, Fort Dodge Animal Health), but these require annual boosting. Several experimental vaccines for humans based upon live-attenuated virus, purified protein, viral vectors, or DNA plasmids are under development (see reviews [19-21]) although none has advanced beyond phase II. In theory, WNV E DIII protein is an attractive target for vaccine development because many strongly protective MAbs (in vitro and in vivo) against flaviviruses, including WNV have been localized to this region DIII (reviewed in [22]). Moreover, previous studies have demonstrated the recombinant WNV DIII is a plausible vaccine candidate when administered as a recombinant protein [23], but less effective when expressed from a DNA plasmid [24]. In this study, we developed candidate WNV DNA vaccines with greater immunogenicity and protection using DIII or truncated E proteins conjugated to the molecular adjuvant P28.

## Materials and methods

### Virus and cell lines

WNV (TX114 strain), isolated from a blue jay in Texas in 2002 was used for all studies except for the passive antibody transfer experiment. The virus was propagated once in Vero cells, aliquotted, and then frozen at -80°C. For the passive antibody transfer experiments, the lineage 1 New York WNV strain 3000.0259 that was isolated in 2000 was passaged once in C6/36 *Aedes albopictus *cells to generate an experimental stock. 293T (human embryonic kidney) and Vero (African green monkey kidney) cells were maintained in DMEM with 10% FBS and 1% penicillin/streptomycin (P/S). Raji cells stably expressing DC-SIGNR were maintained as described [25].

### Construction and expression of DNA vaccine plasmids

**Figure 1:**
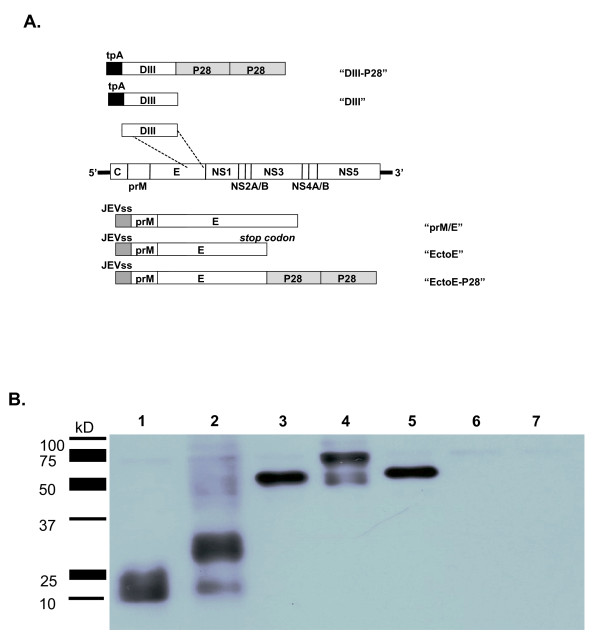
**Schematic diagram of constructs and expression of vaccine plasmids**. **A**. A diagram of the WNV genome is shown in the center, and the segments of the genome used in the vaccine constructs are shown above (DIII-modified) and below (prM/E-modified). The construct expressing prM/E was previously described [26]. The DIII region of the E gene (amino acids 586-705) was cloned downstream of the tpA leader sequence, and in some cases, P28 was also cloned in frame and directly after the 3' end of the DIII gene. An artificial BamHI site and stop codon was engineered at position 705 in the E gene to create the truncated Ecto E gene, and P28 was cloned into the Ecto E construct using the BamHI site to create the Ecto E-P28 construct. **B**. Supernatants from 293T cells transiently transfected with plasmid DNA were assessed by SDS-PAGE and Western blot. The membrane was probed by with the DIII-specific monoclonal antibody, 7H2. Lane 1: DIII-DNA; Lane 2: DIII-P28-DNA; Lane 3: prM/Ecto E-DNA; Lane 4: prM/Ecto E-P28-DNA; Lane 5: prM/E-DNA; Lane 6: P28-DNA only; Lane 7: Vector DNA only.

The WNV prM/E eukaryotic expression vector, pCBWN, has been previously described [26] and encodes the prM and E gene segments (accession number DQ211652) from the strain NY99-6480 strain [26]. To generate an Ecto E DNA vaccine, the glycine residue at position 706 was converted by mutagenesis to a TAG stop codon (Fig. 1A) [27]. The DIII of E (amino acids 296-415) was cloned in frame with the tPA leader sequence in pTR600 (Fig. 1A). A second set of plasmids were constructed to express Ecto E or DIII fused in frame with P28 [18,27]. Each gene sequence encoding for two functional copies of P28 was cloned at the 3' end of Ecto E or DIII using unique restriction endonuclease sites. A BamHI restriction endonuclease site was introduced using site directed mutagenesis immediately 5' to the TAG stop site. A (Gly_4_-Ser)_2 _linker was cloned in between each P28 gene. All DNA vaccine plasmids were amplified in *Escherichia coli*, purified using anion-exchange resin columns (Qiagen, Valencia, CA) and stored at 20°C in dH_2_O. Plasmids were verified by appropriate restriction enzyme digestion and sequencing.

293T cells were transfected with 30 μg of DNA using Lipofectamine 2000 according to the manufacturer's instructions (Invitrogen, Carlsbad, CA). Cell culture supernatants were collected 48 hrs post-transfection. Approximately 1.5% of sample volume was loaded onto a 10% polyacrylamide/SDS gel. The resolved proteins were transferred onto a Immobilon PVDF membrane (Millipore, Temecula, CA) and incubated with a 1:5000 dilution of the WNV specific monoclonal antibody (mAb 8150, Chemicon, Temecula, CA) in PBS containing 0.05% Tween-20 and 5% nonfat dry milk. After extensive washing, bound antibodies were detected using a 1:10,000 dilution of horseradish peroxidase-conjugated goat anti-mouse antiserum, and visualized by chemiluminescence (Western Lightning™, Perkin Elmer, Waltham, MA).

### Virus titrations and immunohistochemistry

All virus titrations were performed on Vero cell monolayers. Briefly, cells were incubated with indicated serial dilutions of virus or mouse serum for 1 hour at 37°C. Subsequently, the virus/serum inocula were removed and replaced with a semi-solid overlay of carboxy-methylcellulose in OptiMEM or 1% low melting point agarose (SeaPlaque) in α-MEM (Invitrogen) supplemented with 3 or 4% FBS. Cultures were incubated for 24-72 hours prior to fixation with a 50:50 v/v mixture of methanol and acetone or 10% formadehyde. For plaque assays, staining was performed with 1% (w/v) crystal violet in 20% ethanol and scored visually on a light box. For focus formation assays, foci were visualized by immunohistochemistry.

Immunohistochemistry was performed on fixed and dried cell monolayers by first rehydrating with 3% FBS in PBS (blocking buffer) for 1 hour, then replacing the media with a monoclonal antibody specific to WNV E protein (7H2; BioReliance Corporation, Rockville, MD) diluted in blocking buffer and incubated for at least 1 h. The primary antibody solution was removed and monolayers were washed thrice in PBS prior to adding the goat anti-mouse secondary antibody conjugated to peroxidase diluted in blocking buffer. After 1 h, monolayers were washed thrice in PBS again. WNV-infected cells were visualized by adding the peroxidase substrate (Enzo Diagnostics, Farmingdale, NY).

### Vaccination and Viral Challenge

Female C57BL/6 mice (n = 5-8 mice per group; aged 6-8 weeks) were purchased from Harlan Sprague Dawley, (Indianapolis, IN, USA), immunized with each DNA vaccine plasmid intramuscularly (IM, 50 μg DNA injection into thigh) or by gene gun (particle bombardment with 2 μg DNA coated on gold bullets) and then boosted with the same dose on weeks 3 and 6. In some cases 0.2 μg or 0.02 μg of vaccine plasmid as a dose response was administered in a mixture of vector plasmid to keep a total of 2 μg total DNA vaccine. Blood was collected from anesthetized mice via the retro-orbital route on weeks 5 and 8 post vaccination, then centrifuged at 6000 rpm for 10 min to separate the serum. Sera were transferred to new vials and frozen at -20°C.

For challenge, naïve or vaccinated mice administered 1000 focus forming units (FFU) of WNV (TX114 strain) in a volume of 0.1 ml by the intraperitoneal route. WNV was diluted in a filtered solution of 10% fetal bovine serum (FBS) in phosphate buffered saline (PBS) prior to the mice infections (diluents). Mice were weighed daily to determine percent weight loss, and monitored to determine the severity of sickness. Moribund mice (severe lethargy, hunched posture and ruffled fur) were euthanized by CO_2 _asphyxiation and recorded as dead for the next day. All mouse experiments were performed in accordance and with approval of the Washington University or University of Pittsburgh Animal Studies guidelines under BSL-3 conditions.

### Passive transfer of antiserum to naïve mice

Five week old C57BL/6 mice (Jackson Laboratories) were infected by subcutaneous route with 10^2 ^PFU of WNV 3000.0259 diluted in Hank's Balanced Salt Solution containing 1% heat-inactivated FBS. For antibody protection studies, one day prior to infection mice were treated by IP injection with indicated amounts of immune (gene gun vaccinated mice) or naïve serum diluted in 100 μl PBS. Mice were monitored daily for 21 days for morbidity and mortality.

### Enzyme-Linked Immunoabsorbant Assay (ELISA)

A quantitative ELISA was performed to assess anti-DIII specific IgG in serum of vaccinated mice. Individual wells of a 96 microtiter plate were coated overnight at 4°C with WNV DIII proteins produced from transfected 293T cells and then blocked (25°C for 2 hr) with PBS supplemented with Tween-20 (0.05%) and nonfat dry milk (5%). Each serum sample was serially diluted and incubated (25°C for 2 hr). Following serial washes with PBS Tween-20 (0.05%), samples were incubated (25°C for 1 hr) with HRP conjugated goat anti-mouse IgG (1:5000) or one of four IgG subclasses (IgG_1_, IgG_2a_, IgG_2b_, or IgG_3_) (Southern Biotechnology, Birmingham, AL) diluted in PBS Tween-20 (0.05%) and nonfat dry milk (5%). Unbound antibody was removed and after additional washes samples were incubated with TMB substrate, and the colorimetric change was measured as the optical density at 405 nm using a plate reader (Biotek Powerwave XS, Winooski, VT USA). The O.D. value of the age-matched naïve sera was subtracted from the OD values of the antisera from the vaccinated mice. Results were recorded as the geometric mean titer (GMT) ± the standard error of the mean (SEM).

### Focus Neutralization Reduction Assay (FRNT)

Sera from individual mice were heat inactivated at 56°C for 30 min. In some cases, sera from moribund or surviving mice following WNV infection were pooled. Pooled sera were diluted 1:10 in DMEM supplemented with 1% FBS, P/S and HEPES, and serially diluted 2-fold thereafter. Pooled naïve sera from uninfected, unvaccinated C57Bl/6 mice were used as a negative control. The 7H2 antibody, which neutralizes WNV in tissue culture [16], was diluted in naïve mouse sera and used as a positive control. Each dilution was incubated in an equal volume of media containing WNV for 1 h at 37°C. The virus-antibody solutions were then placed in duplicate wells in a 24-well plate containing a confluent Vero monolayer and incubated at 37°C for 1 h. Monolayers were rinsed free of unbound virus-antibody solution, rinsed an additional time with PBS, and then covered with the CMC overlay. After 48 hours, monolayers were fixed with a 50:50 v/v methanol and acetone solution. WNV foci were detected by immunohistochemistry as described above. Titer was determined as the dilution in which there was 50% (FNRT_50_) or 80% (FRNT_80_) or greater reduction in the number of WNV foci by immunohistochemical staining.

## Results

### Construction of WNV Vaccine Plasmids

DNA plasmids were constructed that contained either the complete E gene (in context with the precursor viral gene prM "prM/E") or portions of the E gene (ectodomain (Ecto) or domain III (DIII)) (Fig 1A). A second set of plasmids was generated with these same gene sequences conjugated to two copies of the molecular adjuvant P28 to enhance antibody responses to the conjugated antigen (Fig. 1A). All of these gene cassettes were cloned directly downstream of a cytomegalovirus promoter to drive efficient transcription. Each plasmid efficiently expressed the E gene insert in transiently transfected 293T cells as determined by Western blot of clarified cell supernatant with a WNV-specific anti-E MAbs (Fig 1B). DNA plasmids expressing DIII only produced a protein ~10-20 kD in size. The addition of P28 resulted in an expressed protein of ~30 kD (Fig 1B; lanes 1 and 2). Ecto E (~65 kD) and Ecto E-P28 (~70 kD) were efficiently secreted into the supernatants of transiently transfected cells (Fig 1B; lanes 3 and 4). In addition, a 65 kD protein representing E was detected in supernatants from cells transiently transfected with DNA expressing the prM/E gene cassette, which produces subviral particles (SVPs) (Fig 1B; lane 5). As expected, mock-transfected or vector-only transfected cell supernatants showed no reactivity with WNV anti-E MAbs.

### The molecular adjuvant P28 enhances the anti-WNV antibody response

**Figure 2:**
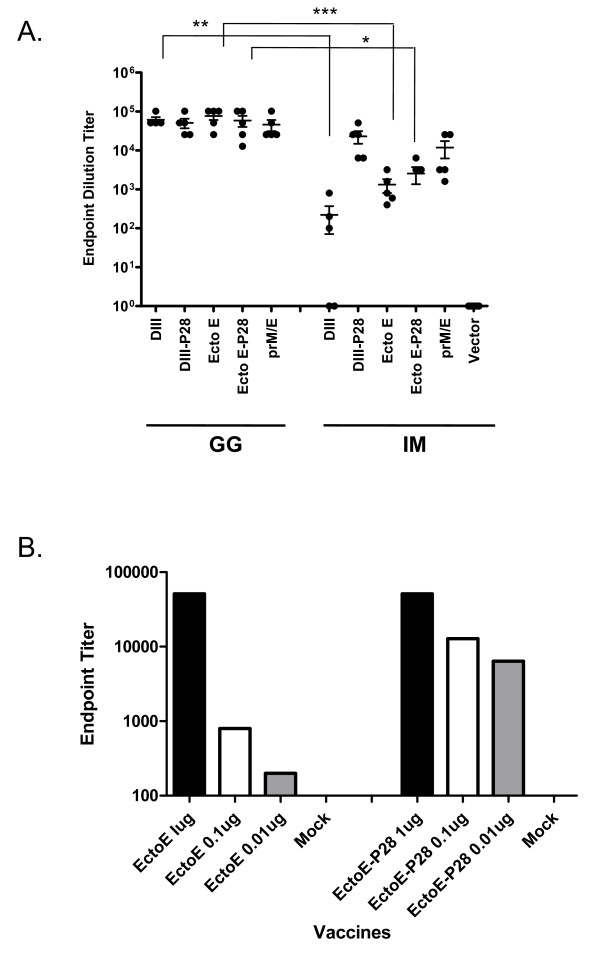
**Vaccine elicited anti-DIII antibodies**. Total IgG titers were measured by ELISA on WNV DIII-coated plates from mice vaccinated ID or IM with DNA plasmids encoding sections of the WNV E gene, with or without molecular adjutant P28 on week 8. Each dot represents an individual mouse. Undetectable antibody titers were arbitrarily assigned a titer of 1. Error bars denote the standard error within the samples with a measurable titer. Representative data from 1 of 2 experiments shown. A 2-way unmatched ANOVA with a Bonferroni post-test was used to determine the significance of the data between groups, which is denoted by asterisks; * P < 0.05, ** P < 0.01, *** P < 0.001.

Mice were vaccinated with the panel of DNA vaccines via one of two routes: gene gun (GG) or intramuscular (IM) at weeks 0, 3, and 6. On week 8, serum samples were collected and the anti-WNV DIII antibody levels were tested by ELISA from individual clarified sera samples (Fig. 2). C57BL/6 mice immunized with all of the DNA plasmids via the gene gun route developed high titers of anti-WNV DIII antibodies. In contrast, mice vaccinated by an intramuscular route with DIII-DNA had significantly lower total IgG titers that were significantly enhanced by conjugation of P28 (Fig. 2A). The enhancement effect was observed following gene gun administration of plasmids only at lower doses of vaccine (Fig 2B). Mice vaccinated IM with Ecto E, Ecto E-P28 or prM/E developed similar titers (Fig 2A).

To characterize further the immune response elicited by these vaccines, the IgG subtypes of the elicited anti-DIII antibodies were determined (Table 1). Gene gun DNA vaccination elicited primarily a T-helper (Th)-2 (characterized by IgG1 isotype), whereas DNA plasmids administered intramuscularly elicited more of a Th-1 response (characterized by IgG2 isotype). C57BL/6 mice immunized by gene gun with DIII- or DIII-P28-DNA elicited predominately IgG_1 _and IgG_2b_. Similar antibody isotypes were elicited with Ecto E and Ecto E-P28 expressing plasmids. Interestingly, the prM/E plasmid elicited a broader IgG isotype profile via both ID and IM routes and included anti-DIII antibodies of the IgG_2c _subclass.

**Table 1:** Anti-DIII Antibody Isotypes

C57BL/6				
Gene Gun	IgG1	IgG2a*	IgG2b	IgG2c
**DIII**	0.44	N.A.	0.18	0.00
**DIII-P28**	0.43	N.A.	0.24	0.00
**Ecto E**	0.41	N.A.	0.19	0.00
**Ecto E-P28**	0.38	N.A.	0.12	0.01
**prM/E**	0.44	N.A.	0.36	0.18
				
**C57BL/6**				
**Intramuscular**	IgG1	IgG2a	IgG2b	IgG2c
**DIII**	0.13	N.A.**	0.01	0.04
**DIII-P28**	0.41	N.A.	0.14	0.02
**Ecto E**	0.23	N.A.	0.08	0.00
**Ecto E-P28**	0.23	N.A.	0.04	0.01
**prM/E**	0.25	N.A.	0.20	0.11

*IgG2a isotype not expressed in C57BL/6 mice.

**N.A. = Not applicable since the IgG2a subtype is not expressed in C57BL/6 mice.

### Protection of mice against lethal WNV challenge

**Figure 3:**
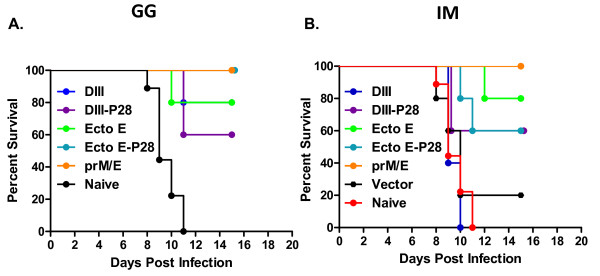
**WNV challenge of vaccinated mice**. Mice that were vaccinated with indicated plasmids were challenged with 10^3 ^FFU of WNV and assessed daily for survival. Mice that were moribund were euthanized and counted as dead the following day. MOCK-challenged mice, which were injected with diluent only, did not die or show signs of illness, and are not shown on the survival curves. The results are pooled from at 5 mice.

**Figure 4:**
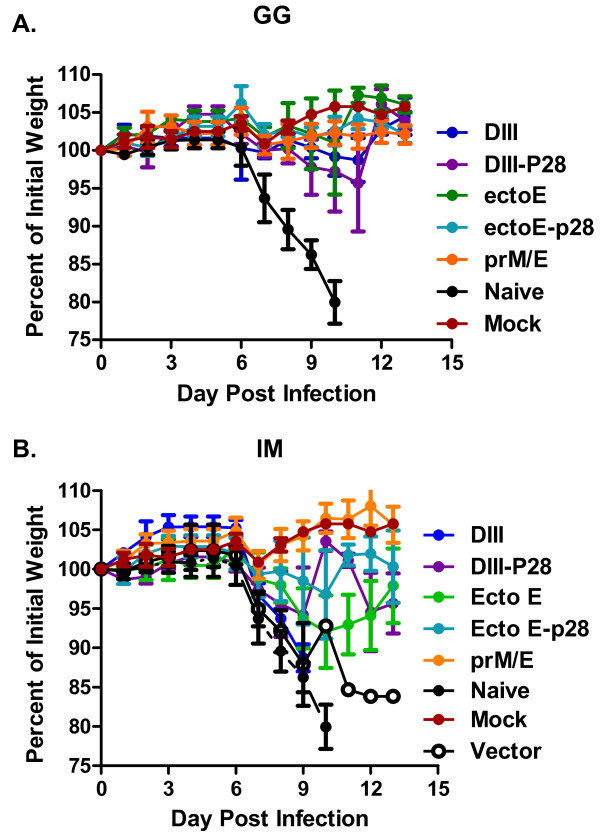
**Weight loss curves of WNV-infected vaccinated mice**. The weights of mice vaccinated by the GG (**A**) and IM (**B**) routes and challenged with 10^3 ^FFU of WNV were recorded daily. Dead and moribund mice were included in the weight loss curves on the day of death, but not after. The daily weight of each mouse was compared to her weight the day of challenge, and data are shown as the average percentage of initial weight for each cohort. The color scheme is identical for both panels. Error bars represent the standard error for all samples available at that time point.

At week 10 (4 weeks from the final vaccination), C57BL/6 mice were challenged with a lethal dose (10^3 ^FFU) of WNV. All unvaccinated and 80% of vector-only vaccinated mice (IM) infected with WNV died by day 11 post-challenge (Fig. 3). The vector-only vaccinated mouse that survived challenge nonetheless showed signs of morbidity (hunchback posture and fur ruffling) and lost ~15% of body weight, but recovered by 1 month post infection (Fig. 4). All mice vaccinated with the prM/E plasmid construct survived lethal challenge (Fig. 3) and did not lose weight, regardless of the vaccination route. In general, mice vaccinated by gene gun had higher rates of survival and less weight loss compared to mice vaccinated intramuscularly. Eighty percent of mice vaccinated ID with DIII-DNA survived challenge with little weight loss (Fig. 3A and 4) whereas, no mice vaccinated IM with DIII-DNA survived challenge (Fig. 3B, p < 0.004). All of these mice lost weight prior to succumbing to infection (Fig. 4C). Conjugation of P28 to DIII enhanced the survival rate to 60% when the DNA was administered IM (Fig. 3B, p < 0.046). Interestingly, the disparity in survival between ID and IM vaccination routes was also apparent in mice vaccinated with Ecto E DNA (Fig. 3). Eighty percent of mice vaccinated ID or IM with Ecto E-DNA survived challenge, and conjugation of P28 to Ecto E increased the survival rate (100%) of ID-vaccinated animals, but slightly decreased rate in IM-vaccinated mice (60%, Fig 3). Although these survival results were not statistically different between these groups, survival did correlated with weight loss, as mice that lost more weight had the highest morbidity and mortality (Fig. 4).

**Figure 5:**
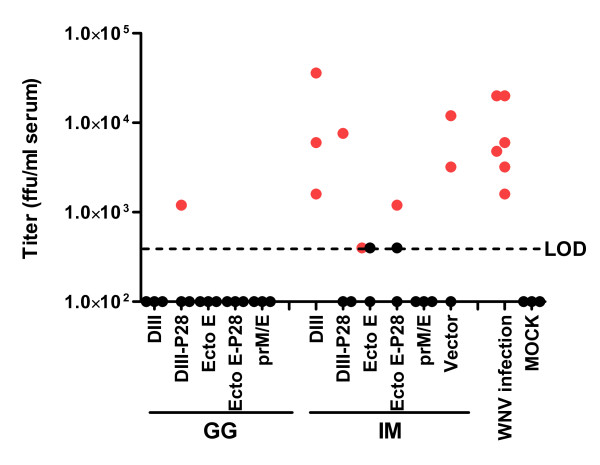
**Viremia of protected and unprotected mice following WNV challenge**. Sera taken from the blood of three mice from each group 2 days after infection were titrated on Vero cells to determine levels of virenia. Each point represents the titer from one mouse; black and red dots denote animals that survived and died, respectively. The dashed line represents the limit of detection (LOD) for this assay, which was 4 × 10^2 ^FFU/ml of serum. Mice with a viremia less than the LOD were arbitrarily shown as dots at the bottom of the graph.

To determine if protection against WNV challenge prospectively correlated with a reduction in viremia, the sera at day 2 post-infection in each vaccine group was analyzed for infectious virus by a focus forming assay. Vaccinated C57BL/6 mice that survived infection (Fig. 5) had viremia that was at or below the limit of detection at day 2 post-challenge. With the exception of one mouse vaccinated with the Ecto E vaccine, all mice that died had a viremia at day 3 of greater than 4 × 10^2 ^FFU/ml (Fig 5).

### Neutralization titers

Sera from mice immunized with the DNA vaccines were assayed for the ability to neutralize WNV infection in cell culture (Table 2). Serum samples collected at week 8 and again at 1 month post challenge (week 14 of study) were divided into two groups: (1) mice that survived subsequent lethal challenge and (2) mice that did not survive challenge (obtained from terminally moribund mice immediately prior to euthanasia). Mice vaccinated with any of the DNA vaccines by gene gun that survived challenge had high neutralizing titers at eight weeks (1/80-1/320; FRNT_80_), whereas those that died from challenge had lower titers <1/20 (Table 2). In contrast to gene gun vaccination, only mice vaccinated with prM-E DNA intramuscularly had high neutralizing titers, which again correlated with survival. Mice vaccinated via gene gun with DNA plasmids expressing DIII, Ecto E, or these immunogens conjugated to P28 had titers <1/20 (FRNT_80_) did not survive infection. Similar results were observed using a FRNT_50_, albeit the titers were higher than FRNT_80_. Regardless of the route of vaccination, mice that survived challenge exhibited an immunological boost by 14 weeks since neutralizing titers rose following infection (FRNT_80_; 1/320-1/1280).

**Table 2:** FRNT and Mouse Survival

Sample	Group	Survival [# pooled/# in vaccinated group]	FRNT50*	FRNT80	FRNT50	FRNT80
GG			Pre-infection		Post-infection	
1	DIII	Yes [4/5]	640	80	2560	320
2	DIII	No [1/5]	<20	<20		
3	DIII-P28	Yes [3/5]	320	80	1280	320
4	DIII-P28	No [2/5]	40	20		
5	ectoE	Yes [4/5]	320	320	2560	640
6	ectoE	No [1/5]	40	<20		
7	ectoE-28	Yes [5/5]	640	320	2560	640
8	prM/E	Yes [5/5]	320	160	1280	640
9	naïve	No [5/5]	20	<20		

IM			Pre-infection		Post-infection	
10	DIII	No [5/5]	<20	<20		
11	DIII-p28	Yes [3/5]	20	<20	1280	640
12	DIII-p28	No [2/5]	20	<20		
13	ectoE	Yes [4/5]	40	20	2560	1280
14	ectoE	No [1/5]	20	<20		
15	ectoE-p28	Yes [3/5]	40	20	2560	640
16	ectoE-p28	No [2/5]	<20	<20		
17	prM/E	Yes [5/5]	320	160	2560	640
18	vector	Yes [1/5]	40	<20	640	80
19	vector	No [4/5]	<20	<20		

* Titers shown as the reciprocal titer

### Passive sera transfer protects mice from virus infection

**Figure 6:**
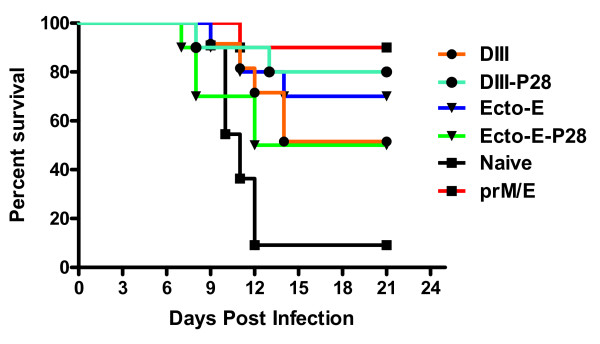
**Passive transfer of antibody from vaccinated mice protects against lethal WNV challenge**. Five week-old C57BL/6 mice were pre-treated with 25 μl of serum from the indicated vaccinated mice or naïve animals at day -1 by intraperitoneal injection. On day 0, mice were infected with 10^2 ^PFU of WNV by subcutaneous route and monitored for survival. All vaccinated mice showed statistically significant protection (P < 0.05, log rank test) compared to mice receiving naïve sera. Data reflect two independent experiments with a total of n = 10 mice per arm.

Although several mice immunized with DIII plasmids survived infection, it remained unclear mechanistically whether this was due to antibodies or possibly, memory CD4^+ ^and CD8^+ ^T cell responses To determine if anti-DIII antibodies alone could afford protection against WNV infection, pooled antiserum from each gene gun vaccinated group was transferred intraperitoneally into naïve 5 week-old C57BL/6 mice, which were then challenged with 10^2 ^PFU of WNV (Fig. 6). Fifty to seventy percent of mice administered sera from mice vaccinated with DIII, Ecto E or Ecto E-P28 survived challenge 21 days post-infection. Sera from mice vaccinated with DIII-P28 or prM-E showed greater (80-90%) survival compared to DIII alone, although this did not attain statistical significance. As expected, nearly all mice (10% survival)) administered naïve sera succumbed to virulent WNV infection.

## Discussion

Although it has been a decade since the emergence of WNV in North America, there remains no effective, licensed vaccine to combat WNV induced disease in humans. Although candidate vaccines have not advanced beyond phase I and II clinical trials for humans [19,28], there are currently approved inactivated and DNA vaccines licensed for use in horses and geese. Since neutralizing antibodies may serve as a primary protective function against challenge [5], recent vaccine strategies have focused on using the ectodomain of E or different domains within E to elicit neutralizing anti-WNV antibodies [23,24,26,29-33]. Recent attention has been focused on DIII as a potential immunogen because structural and functional studies suggest that many protective antibodies against WNV recognize this highly conserved epitopes within this region. Some DIII-specific neutralizing antibodies are particularly potent in blocking viral fusion and escape from the endosome [34,35].

In this study, a series of DNA-based vaccines expressing the full length E, Ecto E or the DIII domain of E were fused to the molecular adjuvant P28 to enhance antibody titers. The addition of P28 to DIII or Ecto E increased the anti-DIII IgG antibody titer in C57BL/6 mice. However, a high anti-DIII antibody titer was not sufficient to completely protect against WNV infection. Mice vaccinated with nearly all gene gun delivered vaccines elicited similar high-titer anti-DIII antibodies, however, only the prM/E and Ecto E-P28 vaccinated mice were completely protected from lethal challenge.

Several studies have previously demonstrated the immunogenicity of the E protein DIII domain [23,29,36-40]. However, immunization of purified recombinant DIII has not consistently elicited high-titer neutralizing antibodies, thereby indicating that the neutralizing epitope may be poorly immunogenic in the context of a soluble protein, or that a dominant non-neutralizing epitope is present on the soluble DIII but is not exposed in the context of the virion. Expression of DIII from a DNA vaccine plasmid also has been less than optimal in eliciting neutralizing antibodies. The results from our study suggest that DIII may be less efficiently secreted from transfected cells (data not shown), which may in part explain the lowered immune responses seen during vaccination with DIII alone. Conjugation of P28 may assist DIII protein secretion from transfected cells, helping to explain why the P28-conjugated vaccines elicited higher DIII-specific antibody titers (by ELISA) and higher protection against lethal challenge than non-conjugated vaccines in some cases. Although the use of molecular adjuvants, such as P28, did not skew the antibody repertoire, they did increase the efficacy of the response of DIII-based DNA vaccines. An analogous increase in overall titer was observed when JEV or WNV DIII was linked to IL-15 [24].

Prospective studies have shown a direct correlation between the level of neutralizing antibody prior to challenge, the magnitude of viremia, and survival rates in mice [5]. Nonetheless, some mice vaccinated IM with DIII-P28, Ecto E, and Ecto E-P28 were protected from challenge despite the absence of high-titer pre-challenge neutralizing antibodies (FRNT_80 _≤ 20). Although further mechanistic studies are required, we suggest three possible explanations: (a) non-neutralizing antibodies are protective through complement and/or Fc┄R-dependent functions. Indeed, we have previously seen this phenotype with mAbs against WNV NS1, which is absent from the virion [41]; (b) the *in vitro *neutralization assay does not accurately reflect possible neutralization of virus *in vivo*. Antibodies that block virus attachment of one cell type (*e.g*., Vero cells) may not function effectively against a second more physiologically relevant cell type (*e.g*., dendritic cells). Of note, differences in neutralization potency among cell types were observed with mAbs against epitopes in DI and DII of WNV E protein [42]; and/or (c) T cell responses to peptide epitopes in the E protein independently contribute to protection. The isotype of the polyclonal antibody in part determines the effector functions of the anti-WNV antibodies and identifies the T helper cell bias (required for antibody class switching). Antibodies of the IgG2a/c and IgG2b subclass fix complement proteins C1q and C3 and can opsoznize and inhibit flavivirus infection [43-45]. IgG2a/c bind FcγRI with high avidity facilitating enhanced uptake of virus-antibody complexes by macrophages. The predominant IgG isotype detected was IgG1 indicating a Th2 bias. However, IgG2b was detected in almost all vaccine groups, with a strong level of this isotype and IgG2c detected in prM/E vaccinated mice (Table 1), which may help to explain the effectiveness of these vaccines. C57BL/6 mice do not express IgG2a, if this isotype was associated with protection, could have been an explanation for the inconsistent protection with these vaccines.

C3d and P28 have been used as effective molecular adjuvants to elicit high titer antibodies against other pathogens [18,27,46-53]. This study extends this platform to WNV, and likely other flaviviruses due to the similarity in E protein structure and function within the genus. Interestingly, gene gun administration of DNA plasmids elicited higher titer antibody responses and broader protection against WNV infection than through the IM route. For mice that were vaccinated gene gun, there was a clear correlation between viral neutralization titers and survival. This correlate was less apparent in IM-vaccinated mice, with most mice surviving infection having low neutralization titers. This discrepancy in survival may be explained, at least in part, by the types of cells that internalize the DNA plasmids and express and/or present these antigens. Muscle cells and infiltrating appear to internalize plasmid DNA following IM administration [54]; these cell types may not efficiently secrete these viral proteins. Gene gun differs from intramuscular or intradermal injection of DNA with a needle and syringe in that it results in direct delivery of the vaccine into the intracellular environment [55]. Gene gun delivery of DNA plasmids is complex and can involve both non-professional antigen presenting cells (APC), such as keratinocytes and professional APCs, such as Langerhans cells [56] and [57]. Compared to other routes of delivery, gene gun inoculations can induce both antibody and CD8^+ ^T cell responses with substantially lower doses of DNA. The effectiveness of this system is likely related to the use of a delivery technology that deposits DNA directly into cells [55]and [57] as well as the immune competence of the epidermis as a delivery site [58] and [59]. Skin cells likely traffic to the draining lymph nodes where the expressed proteins are processed and presented to immune cells [60,61].

Passive transfer studies with serum from vaccinated mice to naïve mice established that antibody generated after immunization was sufficient for protection. The percentage of mice surviving challenge after passive transfer appeared similar to the percentage of vaccinated mice that survived after direct challenge. Although further studies with depleting anti-CD4 and CD8 mAbs are required to precisely evaluate the contribution of T cells to protection in these vaccinated mice, it is noteworthy that for C57BL/6 mice, the immunodominant H-2b T cell epitopes for WNV fall outside of DIII [62-64].

In general, immunogens based upon Ecto E elicited better protective responses than those based upon DIII. Likely, WNV E proteins contain multiple neutralizing epitopes in separate domains and therefore, a broader panel of neutralizing antibodies can be generated. The prM/E plasmid produces SVPs, which are effective immunogens since they contain conformationally relevant prM and E protein [14,65,66]. However, Ecto E-P28 elicited comparable immune responses and protection as prM/E when delivered ID, indicating that DNA vaccination can be as effective as the prM/E vaccines currently used for animal vaccines.

The results from the WNV vaccines described in this report indicate that DIII can be an effective immunogen when expressed from a DNA plasmid, when conjugated to a molecular adjuvant like P28 and delivered as a gene gun based DNA vaccine. The mechanism of delivery could account for the induction of protective responses. Gene gun elicits a T cell helper type 2 (Th2) bias, as indicated by the predominance of elicited IgG_1_, which may be just as an important factor in eliciting neutralizing antibodies as the immunogen epitopes. Nonetheless, our studies have not yet shown that DIII elicits superior neutralizing responses when conjugated to a C3d molecular adjuvant. Although further studies are warranted, we speculate that this is due to the presence of immunodominant non-neutralizing epitopes on the A-B loop that is normally solvent inaccessible [67]. Reverse genetic studies are underway to create DIII variants that lack this immunodominant epitope and thus, focus the immune response on the lateral ridge epitope, which is recognized by highly neutralizing antibodies. By combining this molecular approach with the addition of P28, alone or with other vaccine modalities, we believe it will be possible to create a catalogue of safe immunogens that elicit high-titer neutralizing antibody responses against all flaviviruses.

## Competing interests

The authors declare that they have no competing interests.

## Authors' contributions

MDD and DMC constructed and characterized the vaccines, MDD and SLR performed animal vaccinations, SLR performed virological analysis, and MDD, SLR, MRV, EM immune analysis. MDD, SLR, MSD, and TMR wrote the manuscript. MSD and TMR conceived the studies and participated in experimental design and coordination. All authors read and approved the manuscript.
